# Biologics targeting IL-17 sharply reduce circulating T follicular helper and T peripheral helper cell sub-populations in psoriasis

**DOI:** 10.3389/fimmu.2024.1325356

**Published:** 2024-05-21

**Authors:** Sotirios G. Tsiogkas, Athanasios Mavropoulos, Efthimios Dardiotis, Efterpi Zafiriou, Dimitrios P. Bogdanos

**Affiliations:** ^1^ Department of Rheumatology and Clinical Immunology, Faculty of Medicine, University of Thessaly, Larissa, Greece; ^2^ Department of Neurology, Faculty of Medicine, University of Thessaly, Larissa, Greece; ^3^ Department of Dermatology, Faculty of Medicine, University of Thessaly, Larissa, Greece

**Keywords:** psoriasis, cTfh, cTph, anti-IL-17, PBMCs, secukinumab, brodalumab, psoriatic disease

## Abstract

**Introduction:**

Circulating T follicular helper (cTfh) cells and circulating T peripheral helper (cTph) cells (which share common characteristics with the cTfh population) are implicated in the pathogenesis of immune-mediated and autoimmune diseases such as psoriasis (Ps). Their close interplay with the interleukin 17 (IL-17) axis and the ex vivo effect of IL-17-targeting biologic agents used to treat Ps on them are elusive. This study aimed to investigate the effect of biologics targeting IL-17 on cTfh and cTph cell subpopulations isolated from the blood of patients with Ps.

**Methods:**

Peripheral blood mononuclear cells (PBMCs) were isolated from patients with Ps at treatment initiation and three months later. Samples were also collected from controls. Cells were stained using monoclonal antibodies. Flow cytometry assessed the fraction of cTfh (CD3^+^CD4^+^CXCR5^+^) and cTph (CD3_+_CD4_+_CXCR5_-_PD-1_hi_) cells..

**Results:**

Flow cytometric analysis showed increased fractions of activated cTfh subsets including ICOS^+^ and ICOS^+^PD-1^+^ expressing cells, in patients compared to controls. Biologic blocking of IL-17A diminished the cTfh population. Furthermore, ICOS^+^ and ICOS^+^PD-1^+^ sub-populations were also inhibited. Finally, the cTph cell fraction significantly decreased after three months of successful treatment with biologics.

**Conclusion:**

Early anti-IL-17-mediated clinical remission in Ps is associated with decreased cTfh and cTph cell subpopulations.

## 1 Introduction

Psoriasis (Ps) is an inflammatory disease of the skin. The role of effector T cell populations in the development of Ps has been extensively studied. However, the involvement of follicular helper T (Tfh) cells has not yet been explored in detail. Tfh cells constitute a distinct CD4^+^ T helper (Th) population, identified by B-cell lymphoma 6 (Bcl6) and surface markers such as CXC chemokine receptor 5 (CXCR5) (1), inducible co-stimulator (ICOS) and programmed death 1 (PD-1) protein (2), that supports the proliferation of B cells, and the production of antibodies (3, 4).

Another cell population of relevance, the T peripheral helper (Tph) cell subset, constitutes a CD4^+^ subset with properties and characteristics that resemble those of Tfh-cells. Tph cells are not characterized by CXCR5 expression; on the contrary, PD-1, ICOS, interleukin (IL-) 21 and CXC motif chemokine ligand 13 (CXCL13) molecules are highly expressed (5). Circulating Tph (cTph) cells, phenotypically defined as CD4^+^CXCR5^-^PD-1^hi^ cells (6), specialize in supporting B cells within non-lymphoid tissues. Their participation in the induction of autoimmunity has been supported (7, 8).

Sampling constraints in humans have hindered attempts to understand better the exemplary contribution of Tfh and Tph cells in inflammatory diseases. Researchers have turned to the investigation of circulating Tfh (cTfh) cells, a CXCR5^+^ Tfh-like cell population that may not express Bcl6 but shares immunophenotyping properties and functions with tissue Tfh populations (1). cTph cells have also been studied in peripheral blood in the context of autoimmunity due to restraints of exploring them into inflamed tissues (5).

Recent evidence has implicated both cell subsets in psoriasis. Firstly, cTfh sub-populations and cTph cells increase in the periphery of patients with Ps (9–11). Secondly, expanded populations are accompanied by elevated activation levels as defined by the expression of ICOS or PD-1 (12–14). Thirdly, cTfh subpopulations correlate with Ps severity (10, 11, 13). Finally, cTfh have been observed to format ectopic lymphoid-like structures, which serve as starting points for various immune interactions and advancement of pathogenic disease-specific cell populations (4, 15). In psoriatic skin, such clusters of dendritic and T-cell subsets have been recognized in developing pro-inflammatory conditions that mediate skewing towards Th17 and keratinocyte proliferation (16–18). Interestingly, cTfh cells have been identified in increased numbers in psoriatic skin (13).

Recent studies have identified IL-17 as a critical molecule for the Tfh population. IL-17 has been found to arrest B cell migration, thus promoting the formation of germinal centers in which Tfh and B cell interactions occur (19). Significantly, *in vitro* IL-17 blockade has been found to suppress plasmablast differentiation (20). In a murine model of experimental autoimmune encephalomyelitis, myelin-specific Tfh could not induce disease when transferred into recipient mice; however, anti-CXCL13 treatment (CXCL13: chemokine that binds to CXCR5, which is highly expressed on Tfh) attenuated Th17-mediated disease severity (21).

Since patients with Ps are frequently treated with IL-17-neutralizing agents, and an association between increased expression of molecules within the IL-23/IL-17 pathway and enhanced Tfh-mediated functions in mice was recently reported (20), we explored the effect of anti-IL-17 biologic treatment on human cTfh cells. The current study aimed to assess by multicolor flow cytometry the impact of biologics specifically targeting the IL-17 axis (secukinumab or brodalumab) on the fractions of cTfh and cTph cell subsets in the blood of patients with Ps.

## 2 Patients and methods

### 2.1 Patients

Thirty consecutive individuals with a diagnosis of moderate-severe Ps vulgaris, as defined by a Psoriasis Area and Severity Index (PASI) of more than 7, who attended the Department of Dermatology of University General Hospital of Larissa in Central Greece were characterized as eligible for enrollment. Patients with no other autoimmune or autoinflammatory comorbidity were enrolled. Blood collection from Ps patients was attained at baseline and three months of therapy and prior to therapy initiation, a 3-month washout period had preceded. During treatment, patients did not receive any other systemic drug for the management of Ps. Ten demographically matched HCs were also enrolled. Clinical descriptions of the cohorts enrolled are presented in **Table 1**. PASI improvement at three months determined response to therapy. A ≥50% PASI improvement was defined as PASI50, a ≥90% PASI improvement as PASI90, and a 100% improvement as PASI100.

**Table 1:** Demographics of psoriasis patients and healthy controls.

Parameter	Psoriasis Patients (n=30)	Healthy Controls (n=10)
Age (years)	53.5 ± 10.7	48.5 ± 11.4
Gender (M/F)	17/13	6/4
BMI (kg/m^2^)	27.3 ± 3.21	24.5 ± 3.4
PASI (Baseline/After)	(14.7 ± 6/1.73 ± 1.86)	–
Age of disease onset (years)	40.1 ± 11	
Previously treated* with csDMARD	7/30	
Previously treated* with apremilast	5/30	
Previously treated* with biologic anti-TNF	3/30	
Previously treated* with biologic anti-IL12/23	3/30	
Naïve to previous treatment	12/30	
PASI50	30/30	
PASI75	26/30	
PASI90	21/30	
PASI100	7/30	

csDMARD, conventional synthetic disease-modifying antirheumatic drug; PASI- 50, 75, 90, 100: a 50%, 75%, 90%, 100% improvement in Psoriasis Area Severity Index respectively, *refers to last treatment received before anti-IL-17 biologic treatment initiation.

From each study participant, a peripheral blood sample of 30 mL was collected and used to obtain peripheral blood mononuclear cells (PBMCs) utilizing a standard protocol as previously described (22). Briefly, blood layering on the surface of a LymphoPrep gradient (Axis-Shield, Oslo, Norway), centrifugation, and washing (twice) with serum-free RPMI 1640 (Pan Biotech, Aidenbach, Germany) were followed to accomplish PBMC isolation. A hemocytometer was used for cell counting. We then assessed cell viability using trypan blue, which consistently exceeded 95%. Afterward, we resuspended PBMCs in a solution containing cell cryoprotectants [dimethyl sulfoxide (10%) and fetal bovine serum (90%)]. PBMCs were subsequently portioned into cryogenic tubes, placed at -80°C for 24 hours, and finally reserved in tanks containing liquid nitrogen until experimental use.

### 2.2 Leukocyte immunophenotyping by multicolor flow cytometry

Leukocyte immunophenotyping was executed as previously described (23). In brief, cryovials containing cells were transferred in the water bath at 37°C and held on the water’s surface for approximately 1 min. until a small portion of ice remained frozen. Then, vials were immediately transferred to the biosafety hood, where pre-warmed RPMI 1640 was added dropwise in each cryotube. Consequently, the content of each cryotube was transferred dropwise in a centrifuge tube. PBMCs that were thawed were centrifuged for 5 min. Moreover, the supernatant was then discharged. Finally, PBMCs were resuspended in a culture medium containing 10% fetal calf serum (FCS) and 90% RPMI 1640. After PBMC thawing and washing, cells were resuspended using a solution containing phosphate buffered saline (PBS) and FCS (2%) (staining buffer). To assess surface phenotypes of isolated PBMCs, we utilized anti-human monoclonal antibodies (mAbs): fluorescein isothiocyanate (FITC)-conjugated anti-CXCR5 (clone J252D4), phycoerythrin (PE)-conjugated anti-PD-1 (clone EH12.2H7), PE-conjugated anti-CCR6 (clone G034E3), peridinin chlorophyll protein (PerCP)-conjugated anti-CD3 (clone HIT3a), PE-Cy7-conjugated anti-CD4 (clone RPA-T4) and APC-Cyanine7-conjugated anti-ICOS (clone C398.4A) (BioLegend, San Diego, USA) (**Supplementary Table 1**). Subsequently, a 30-minute incubation with mAbs on ice was performed, and fixation of PBMCs using a paraformaldehyde solution (2%) was achieved. A supplemental panel was utilized to assess an intracellular marker. Specifically, PBMCs were stained with PE-conjugated anti-human Bcl-6 (clone 7D1), FITC-conjugated anti-CXCR5 (clone J252D4), PerCP-conjugated anti-CD3 (clone HIT3a), and PE-Cy7-conjugated anti-CD4 (clone RPA-T4), as described above. Intracellular staining was carried out following surface staining and fixation. Per the manufacturer’s instructions, a transcription factor buffer set was used (BioLegend, San Diego, USA). To assess apoptosis of PBMCs after thawing for each sample included, we further utilized FITC-conjugated Annexin V using the manufacturer’s protocol that includes Annexin V Binding Buffer (BioLegend, San Diego, USA). A benchtop flow cytometer was used to analyze (Guava EasyCyte 8, Merck-Millipore, Missouri, USA). **Supplementary Table 2** shows the flow cytometer setup used to perform experiments. Logarithmic amplification was applied. Total lymphocytes were gated based on forward and side light scatter. Proper quantification of scarce cell subsets was ensured by collecting ≥3x10^5^ events in each total lymphocyte gate. Isotype controls were utilized to exclude false positive or high background readouts (**Supplementary Figure 1**). To assess the reproducibility of the results, we conducted experiments in duplicate. Data were acquired using guavaSoft software (Merck-Millipore, Missouri, USA) and analyzed in FlowJo (BD Biosciences).

### 2.3 Statistical analysis

Study sample characteristics were described using descriptive statistics. Epitope expression on cell surfaces was reported as a percentage of cells for each epitope. Cell fractions expressing each epitope were presented by mean and standard deviation. Cell-frequency differences between Ps patients and healthy controls (HCs) were assessed using an unpaired t-test or Mann-Whitney U test. Comparisons between two time points for each cell group were executed by paired t-test or Wilcoxon signed-rank test, whichever was appropriate. Shapiro-Wilk test was performed to assess normality. Differences in mean cell percentage changes between various responding groups were explored using one-way ANOVA. Correlations were investigated using the Pearson correlation coefficient. Mean value and 95% confidence interval or median value of difference are reported to describe differences between each participant group or time points of each cell subset. Significance was defined as p ≤0.05. GraphPad Prism software was utilized to perform statistical calculations.

## 3 Results

### 3.1 Clinical characteristics

Lymphocytes and sub-populations were gated as depicted in **Supplementary Figure 2**. **Supplementary Table 3** summarizes cell subset identification—based on immunophenotyping characteristics—used in our research.

After three months of biologic therapy, all Ps patients (30 of 30, 100%) were at least partially responders and, as such, were included in the analyses (21 of 30 Ps patients [70%] were complete responders). There was no significant difference in lymphocyte populations between baseline and post-treatment assessment, as indicated by routine laboratory values (**Supplementary Table 4**). Furthermore, changes in CD3^+^CD4^+^ cell percentages were not observed between baseline and post-treatment (**Supplementary Figure 3**).

### 3.2 Increased fractions of ICOS^+^ and ICOS^+^PD-1^+^ cTfh cells in patients with Ps compared to controls

**Figure 1:**
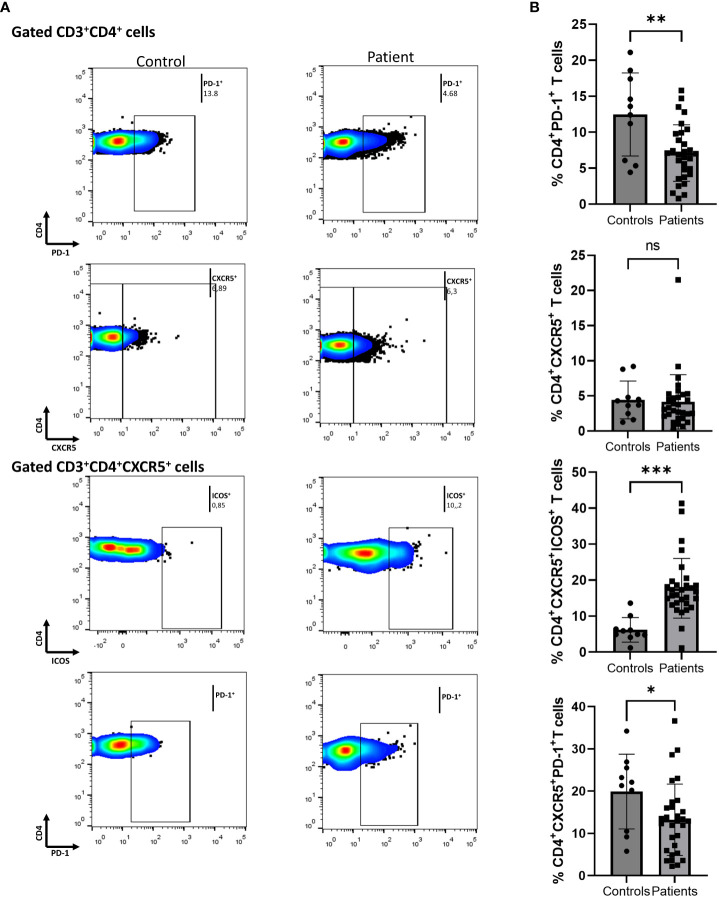
Comparison of CD4^+^PD-1^+^, CD4^+^CXCR5^+^, CD4^+^CXCR5^+^ICOS^+^, and CD4^+^CXCR5^+^PD-1^+^ cell populations between psoriasis (Ps) patients and healthy controls (HCs). A statistical analysis of data derived from plots generated by flow cytometry of PBMCs was performed. Characterization of sub-populations was executed based on CD3, CD4, CXCR5, ICOS and PD-1 surface markers expression. **(A)** Differences in frequencies of CD4^+^PD-1^+^, CD4^+^CXCR5^+^, CD4^+^CXCR5^+^ICOS^+^, and CD4^+^CXCR5^+^PD-1^+^ cell populations between Ps patients and HCs, as presented in representative plots. **(B)** Graphical representation of significant differences in percentages of CD4^+^PD-1^+^, CD4^+^CXCR5^+^ICOS^+^, and CD4^+^CXCR5^+^PD-1^+^ cell subsets between Ps patients (n=30) and HCs (n=10). Graphs, mean ± SD; ns p >0.05; *p ≤0.05; **p ≤ 0.01; ***p ≤0.001.

We initially assessed differences between Ps patients and HCs. To determine the immune-silencing capacity of each subset, we investigated surface PD-1. PD-1 expression was lower in the CD4^+^ compartment in Ps patients (difference between means = -5.4, 95% CI -8.65 to -2.1, p = 0.0020) (**Figure 1**). The percentage of cells expressing ICOS in the same compartment did not differ between the two groups.

Next, we evaluated the differences within the cTfh cell population. We found an increase in the fraction of ICOS^+^ cells in Ps patients compared to controls (difference between means = 11.55, 95% CI 6.06 to 17.04, p = 0.0001) (**Figure 1**). Since an increase in cell activation in disease was observed, we also explored the double positive cTfh subset. The percentage of CD4^+^CXCR5^+^PD-1^+^ICOS^+^ cells was also significantly increased in Ps (difference between means = 3.81, 95% CI 1.56 to 6.07, p = 0.0016). On the contrary, we observed a smaller percentage of PD-1^+^ cells within the cTfh compartment in Ps patients (difference between means = -6.66, 95% CI -12.98 to -0.34).

### 3.3 Biologic blocking of IL-17A diminished the cTfh population in Ps patients

**Figure 2:**
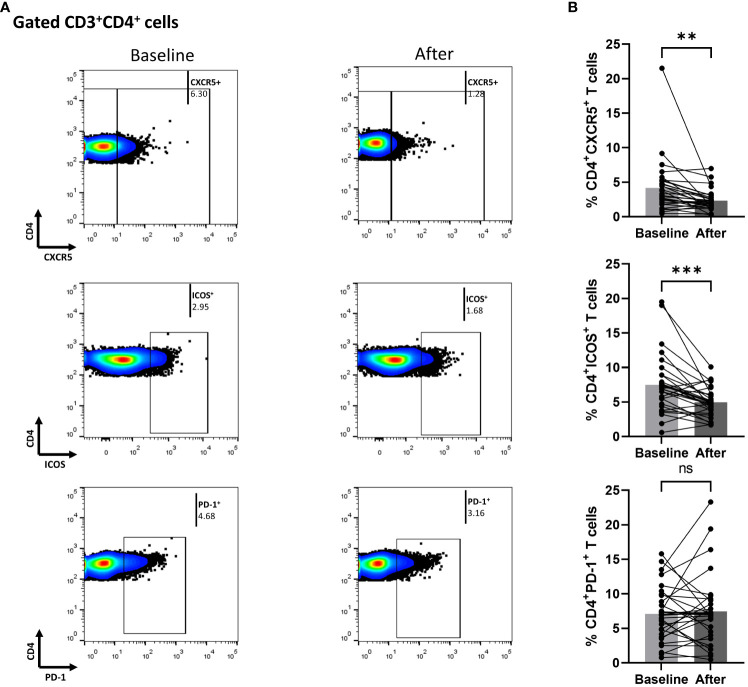
Anti-IL17 treatment significantly decreases percentages of CD3^+^CD4^+^CXCR5^+^ and CD3^+^CD4^+^ICOS^+^ cell populations in psoriasis (Ps) patients. A statistical analysis of data was performed for patients with Ps at IL-17A biologic treatment initiation (baseline) and three months afterwards. Characterization of sub-populations was executed based on expression of CD3, CD4, CXCR5, ICOS and PD-1 surface markers. **(A)** Decreased percentages of CD3^+^CD4^+^CXCR5^+^ and CD3^+^CD4^+^ICOS^+^ cell populations in Ps patients after treatment, as presented in representative plots. **(B)** Graphical representation of significant inhibition of the percentages of CD3^+^CD4^+^CXCR5^+^ and CD3^+^CD4^+^ICOS^+^ cells in Ps patients (n=30) after anti-IL17A biologic therapy. Graphs, mean ± SD; ns p >0.05; **p ≤ 0.01; ***p ≤0.001.

In **Figure 2**, we demonstrate that secukinumab or brodalumab administration, at three months of treatment, significantly inhibited fractions of cTfh (mean of differences = -1.84, 95% CI -3.1 to -0.57, p = 0.0059) and ICOS-producing CD4^+^ T (mean of differences = -2.52, 95% CI -3.78 to -1.27, p = 0.0003) but did not alter the PD-1-producing CD4^+^ cells.

### 3.4 Biologic blocking of IL-17A diminished activated cTfh cells in Ps patients

**Figure 3:**
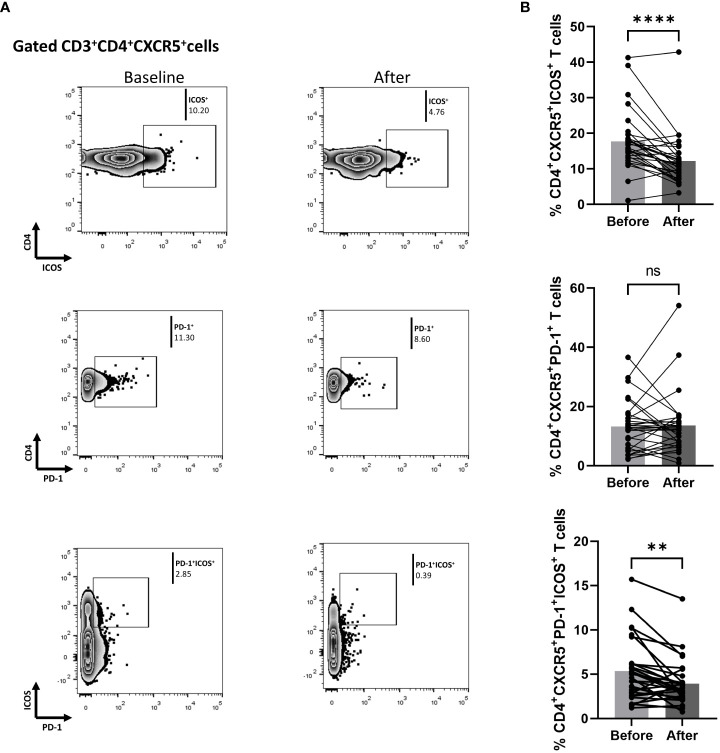
Biologic treatment with anti-IL-17 blocking agents diminishes the frequency of activated cTfh cell populations in psoriasis (Ps) patients. We performed statistical analysis of data for patients with Ps at anti-IL-17A biologic treatment initiation (baseline) and three months afterwards. Characterization of sub-populations was executed based on CD3, CD4, CXCR5, ICOS, and PD-1 surface markers expression. **(A)** Decreased frequency of the CD3^+^CD4^+^CXCR5^+^ICOS^+^ and CD3^+^CD4^+^CXCR5^+^PD-1^+^ICOS^+^ cell populations in Ps patients after treatment, as presented in representative plots. **(B)** Graphical representation of significant inhibition of percentage of the CD3^+^CD4^+^CXCR5^+^ICOS^+^ and CD3^+^CD4^+^CXCR5^+^PD-1^+^ICOS^+^ cell populations in Ps patients (n=30) after anti-IL-17A biologic therapy. Graphs, mean ± SD; ns p >0.05; **p ≤ 0.01; ****p ≤ 0.0001.

We also measured biological treatment-induced changes within the cTfh cell population at three months. As shown in **Figure 3**, the fraction of ICOS-producing cells significantly decreased [mean of differences = -5.5, 95% CI -7.76 to -3.26, p < 0.0001). Interestingly, biological therapy also considerably reduced the proportion of CD4^+^CXCR5^+^PD-1^+^ICOS^+^ T cells (mean of differences = -1.43, 95% CI -2.28 to -0.58, p = 0.0018)]. The proportion of cells expressing PD-1 in the CD4^+^CXCR5^+^ T compartment was also assessed, but no significant difference was observed between the baseline and the post-treatment timepoints. Differences in mean changes between complete and partial responders were not observed for any T cell subpopulation (**Supplementary Figure 4**).

Given the central role of Th17 in the pathogenesis of Ps, we also investigated whether biologic therapy affected the proportion of CD4^+^CXCR5^+^CCR6^+^ cells, but no significant difference was observed (n = 5, mean of differences = 1.15, 95% CI -4.53 to 7.60).

### 3.5 Biologic blocking of IL-17A diminished cTph cells in Ps patients

**Figure 4:**
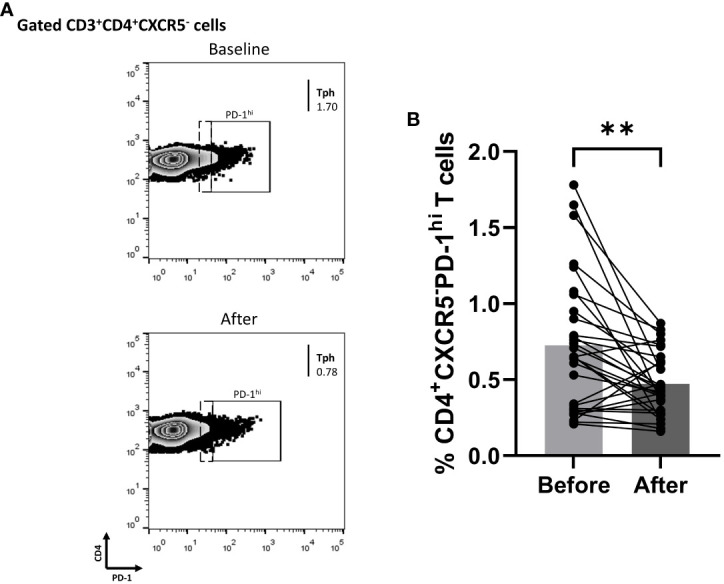
Anti-IL-17 treatment significantly decreases the percentage of CD3^+^CD4^+^CXCR5^-^PD-1^hi^ (Tph) cell population in successfully treated patients with psoriasis (Ps). We performed statistical analysis of data for patients with Ps at IL-17A biologic treatment initiation (baseline) and three months post-treatment. Characterization of sub-populations was executed based on CD3, CD4, CXCR5, and PD-1 surface markers expression. **(A)** Decreased frequency of CD3^+^CD4^+^CXCR5^-^PD-1^hi^ (Tph) cell population in Ps patients after treatment, as presented in representative plots. **(B)** Graphical representation of significant inhibition of the percentage of CD3^+^CD4^+^CXCR5^-^PD-1^hi^ (Tph) cells in Ps patients (n=30) after anti-IL-17A biologic therapy. Graphs, mean ± SD; **p ≤ 0.01.

Since biologics reduced the fraction of blood cTfh cells in Ps patients, we explored whether the percentage of cTph cells was also affected. Indeed, the proportion of Tph in the periphery of Ps patients was significantly decreased (mean of differences = -0.25, 95% CI -0.40 to -0.11, p = 0.0016) at three months of treatment (**Figure 4**).

### 3.6 Significant correlations between clinical improvement and changes in mean percentages of cTfh cell sub-populations were not observed

Significant correlations were not observed between mean changes in PASI scores (baseline versus three months timepoint) and changes in mean percentages of T cell populations (in the corresponding timepoints). Furthermore, significant correlations between PASI scores and the percentages of CD3^+^CD4^+^CXCR5^+^ cells were also not observed in either baseline or the three-month post-treatment timepoint. Of the 30 patients, 17 were treated with secukinumab, and 13 were treated with brodalumab. No significant differences in mean cell percentage changes were observed between secukinumab- or brodalumab-induced effects (**Supplementary Figure 5**). In agreement with our previous findings [22], our cell-thawing protocol did not influence cell viability. Thus, the percentage of apoptotic cells was insignificant (**Supplementary Figure 6**). Assessment of Bcl-6 expression on cell sub-populations showed an 8-fold higher expression of Bcl-6 in the CD4^+^CXCR5^+^ T compartment compared to the CD4^+^CXCR5^-^ (n=5, mean of differences = -1.90, 95% CI -2.67 to -1.12, p = 0.0024, **Supplementary Figure 7**). It should be noted that even in the CD4^+^CXCR5^+^ sub-population, Bcl-6 expression was shallow (n=5). Anti-IL-17 mediated changes in Bcl-6 expression in the CD4^+^CXCR5^+^ compartment were not significant.

## 4 Discussion

In this study, we assessed differences between Ps patients and HCs in the circulating frequencies of cTfh cells, of PD-1-expressing Th and cTfh cells, ICOS-expressing Th and cTfh cells, and ICOS^+^PD-1^+^ cTfh cells.

While some data in the past indicate increased percentages of CD4^+^CXCR5^+^ T cells in Ps (11, 13) our study failed to report them. This is consistent with another study that also reported no distinction between the two groups (12). Such discrepancies could be attributed to differences in patient characteristics, patient ethnic origins, overall PASIs, and previous exposure to therapeutic agents between cohorts included in different studies.

We report that PD-1 expression within the CD4^+^CXCR5^+^ T compartment is reduced in Ps. Inhibited expression of PD-1 in cTfh of Ps patients is likely associated with decreased immune-silencing properties. The concept of PD-1-PDL-1 pathway disruption in Ps has been studied in murine models. For instance, PD-1 knock-out mice presented more significant epidermal hyperplasia and Th17-cytokine expression when exposed to imiquimod compared to wild-type mice (24). Interestingly, the eruption of psoriasiform lesions has been reported in patients with malignancies treated with nivolumab, a human anti-PD-1 antibody (25, 26). Our data are in accordance with previous findings in Ps (12). Regarding the expression of PD-1 in the overall CD4+ T cell population, it should be noted that PD-1 expression can often be an activation marker rather than a marker of cell exhaustion. Accordingly, contradictory results have been shown for the frequency of PD-1^+^ CD4^+^ T cells in patients with psoriatic arthritis compared to HCs (27–29), with some of them reporting decreased PD-1^+^ T-cell percentages (14). All studies, including our own, included a relatively limited number of patients. We also explored levels of cell activation. The ICOS molecule has been known to increase in activated CXCR5+ T cells (30) and has been used as a marker for cell activation (13). Interestingly, blood cTfh cells remain in a non-ICOS-expressing resting state; however, in disease, they are activated and produce ICOS (31). Our results clearly document that ICOS^+^-CD4^+^CXCR5^+^ T cells increase in the blood of patients, which is consistent with published data (11). We also observed an increase in the percentage of double-positive activated cTfh cells (CXCR5+PD-1+ICOS+) in patients with Ps.

After studying the differences between patients with Ps and HCs, we aimed to investigate the effects of anti-IL-17 biologic treatment. Brodalumab and secukinumab significantly reduced the number of cTfh population in the periphery of responding patients with Ps. They also hindered the proportion of activated cTfh cell sub-populations. Specifically, ICOS^+^PD-1^+^-cTfh and ICOS^+^-cTfh cells inhibited post-treatment. In addition, ICOS expression also decreased in total CD4^+^ T cells. Our results clearly indicate post-treatment attenuation of pro-inflammatory stimulatory cell sub-populations, which may contribute to the improvement of the clinical manifestations.

The question of whether the decrease of activated cTfh subsets observed after therapy is a result of the gradual resolution of inflammation or a direct effect of the antibodies on the cTfh cell development pathway (or potentially both) remains. An answer is challenging to obtain in translation studies based on biological material. However, the immunomodulatory properties of IL-17 on Tfh cells have recently been explored in murine models. Indeed, in IL-17 deficient Roquin^san/san^ mice, activated Tfh (CD4^+^CXCR5^+^ICOS^+^PD-1^+^) cells within germinal centers significantly decreased compared to non-deficient mice. Moreover, IL-17 deficiency affected germinal center formation in murine spleens and hindered differentiation towards plasma cells in Roquin^san/san^ mice (32). The addition of neutralizing antibodies against IL-17A in co-cultures of Tfh and B cells has been found to suppress cell differentiation and autoantibody secretion (20). Our data suggest that direct associations between the IL-17 pathway and cTfh cell function exist in humans and in particular in patients with Ps.

In addition, we report that cTph cells decreased after secukinumab or brodalumab treatment. While Ps has been suggested to implicate autoimmune-related mechanisms, few autoantigens, such as cathelicidin, LL37, ADAMTSL5, lipid antigen PLA2G4D, and keratin 17 have been reported (33), but their relevance still remains obscure. The recent identification of Ps-related autoantigens has raised the expectation that B cells may also participate in disease pathogenesis (34, 35), but the extent of such involvement and the relevance to disease progression still remains incomplete. Although antigen-specific T cells have been found in Ps patients, B cell participation and antibody production have also been suggested to engage as potent mechanisms (35). Tph cells have been reported to induce B cell functions in peripheral inflamed tissue, partially in an IL-21-mediated manner. Interestingly, IL-21 belongs to the key molecules driving keratinocyte proliferation and T effector cell differentiation in Ps (36). Interestingly, cTph cells have been reported to correlate with disease severity in Ps (10). Whether the cTph subset participates in disease development through IL-21 production or B cell promotion needs meticulous investigation.

To the best of our knowledge, our study is the first to demonstrate that IL-17 therapeutic biologic blockade modulates cTfh and cTph cell populations and expression of activation markers in Ps patients. Our data document significant differences between patients and controls, as well as substantial changes in cell percentages before and after biological treatment. However, due to the endogenous limitations of our study’s design, we urge caution in interpreting our findings. Our experiments were performed on cryopreserved PBMCs. While it has been reported that functional T cell populations remain stable after long-term cryopreservation (37) and assessment of the percentages of Tfh cells in patients has previously been reported in such cells (38), that kind of manipulation may fundamentally affect cell activation. The findings must be explored further in more extended cohorts for longer periods of time. Nevertheless, this first set of results suggesting that a clinical remission mediated by anti-IL-17 biologics is modulating cTfh cells in the periphery of Ps patients can be the impetus for subsequent investigations.

## Data availability statement

The raw data supporting the conclusions of this article will be made available by the authors, without undue reservation.

## Ethics statement

The studies involving humans were approved by Ethics Committee of the University General Hospital of Larissa, University of Thessaly (#20931-11/06/2021). The studies were conducted in accordance with the local legislation and institutional requirements. The participants provided their written informed consent to participate in this study.

## Author contributions

ST: Data curation, Formal Analysis, Investigation, Methodology, Visualization, Writing – original draft. AM: Data curation, Formal Analysis, Investigation, Methodology, Writing – review & editing. ED: Writing – review & editing, Methodology, Supervision. EZ: Writing – review & editing, Supervision, Conceptualization, Data curation, Investigation. DB: Conceptualization, Supervision, Writing – review & editing, Data curation, Investigation, Funding acquisition, Project administration, Validation, Writing – original draft.
