# Supplementary material for: Biologics targeting IL-17 sharply reduce circulating T follicular helper and T peripheral helper cell sub-populations in psoriasis
Source: Front Immunol. 2024 May 21;15:1325356. doi: 10.3389/fimmu.2024.1325356 (PMC11148216; doi:10.3389/fimmu.2024.1325356)
Supplement: Supplementary file 1 [file DataSheet_1.docx]

Supplementary Material

# Supplementary Figures and Tables

## Supplementary Figures

**
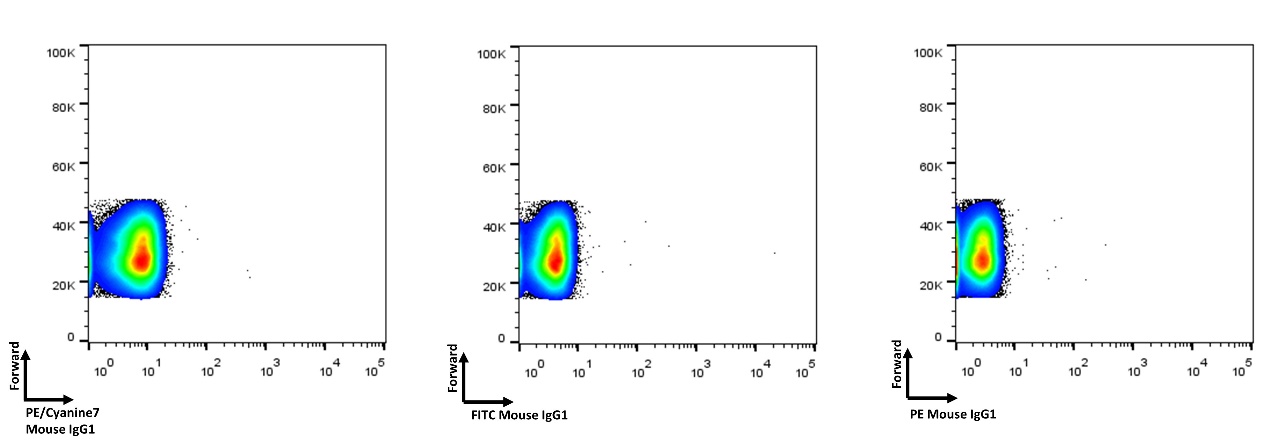
**

**Supplementary Figure 1.** **Isotype controls were utilized to exclude false positive or high background readouts.** Appropriate isotype control antibodies were purchased from BioLegend. To differentiate non-specific background signal FITC Mouse IgG1, κ Isotype Ctrl (FC) (FITC-CXCR5), PE Mouse IgG1, κ Isotype Ctrl (PE-PD-1), APC/Cyanine7 Armenian Hamster IgG Isotype Ctrl (APC/Cyanine7-ICOS), and PE/Cyanine7 Mouse IgG1, κ Isotype Ctrl (PE-Cy7-CD4) were utilized.

##
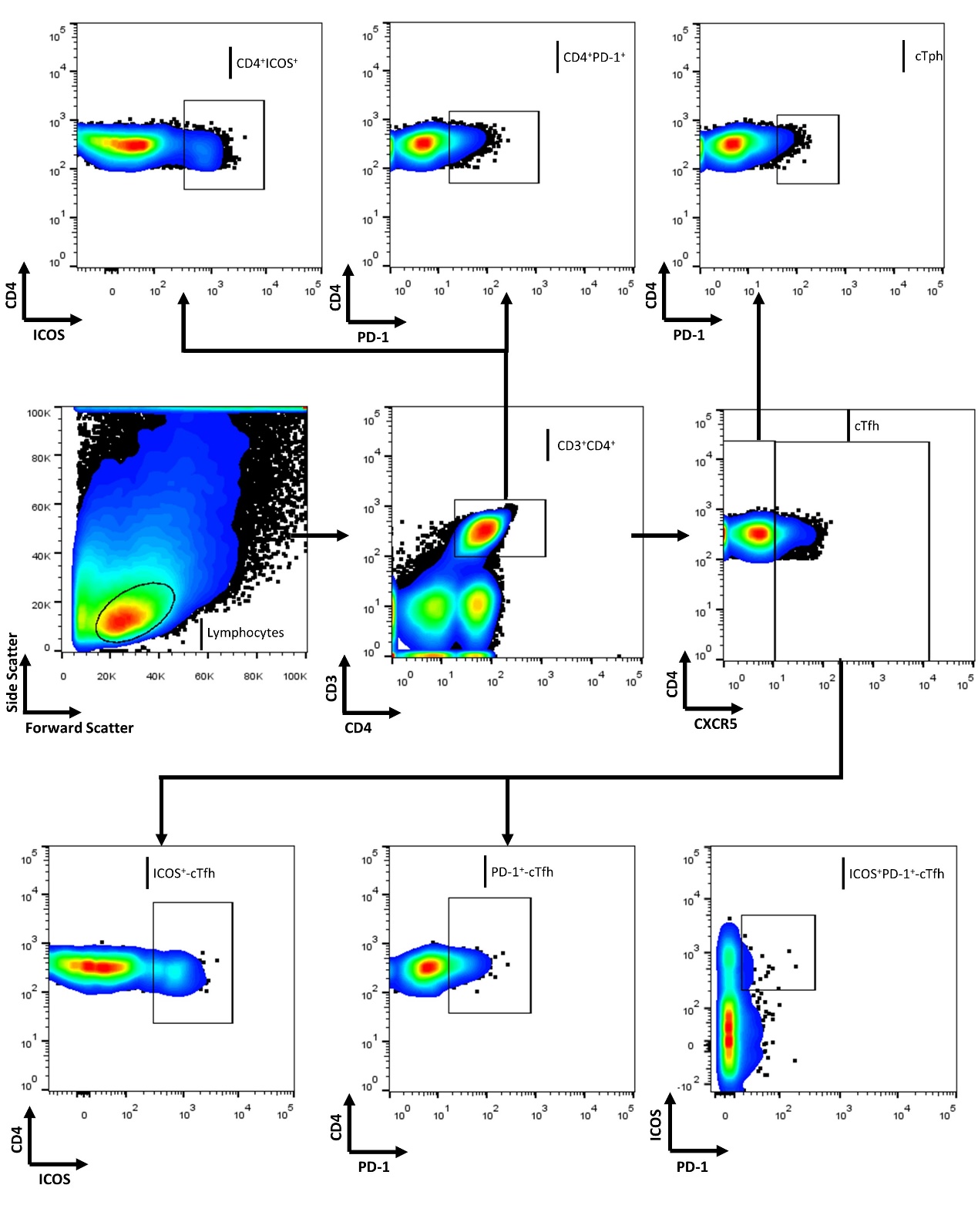


**Supplementary Figure 2.** Consecutive gating of PBMC populations, as depicted in plots generated by flow cytometry, until subsets of interest are properly gated. First, total lymphocytes were gated based on forward and side light scatter characteristics. Consequently, CD3^+^CD4^+^ lymphocytes were gated, and within that population expression of CXCR5, ICOS, and PD-1 surface markers was assessed (in cell percentages). Furthermore, in CD3^+^CD4^+^CXCR5^+^ lymphocyte subset, percentage of ICOS^+^ (ICOS^+^-CD3^+^CD4^+^CXCR5^+^), PD-1^+^ (PD-1^+^-CD3^+^CD4^+^CXCR5^+^) and PD-1^+^ICOS^+^ (activated cTfh) was evaluated, while in CD3^+^CD4^+^CXCR5^-^ lymphocyte subset, proportion PD-1^hi^ cells (Tph) was explored.


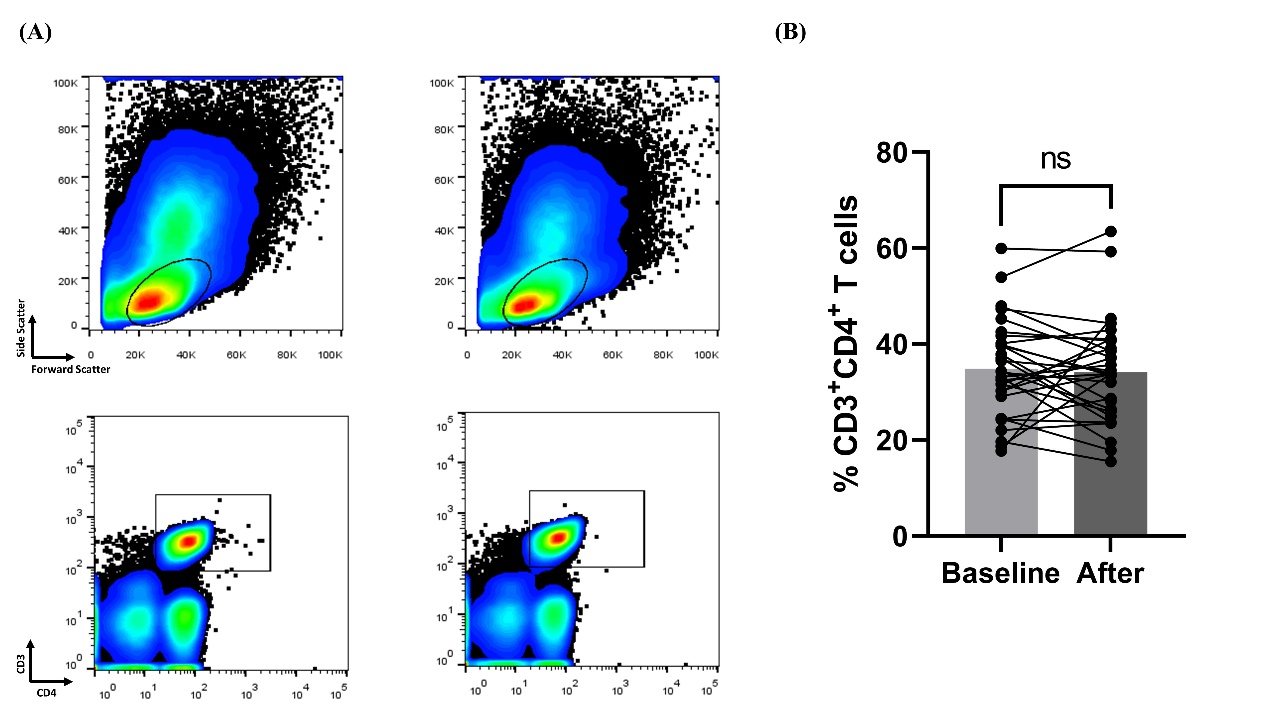


**Supplementary Figure 3.** **CD3^+^CD4^+^ cell population is not affected by anti-IL-17 biologic therapy and frequencies remained relatively unchanged between baseline and after treatment initiation.** **(A)** Relatively unchanged fractions of CD3^+^CD4^+^ T cell populations at baseline and at 3 months of biologic treatment in patients with PS, as presented in representative flow cytometry plots. **(B)** Box graphical representation showing no significant difference in mean CD3+CD4+ cell subset percentage before and after biologic treatment. ns p >0.05, by paired t-test

**
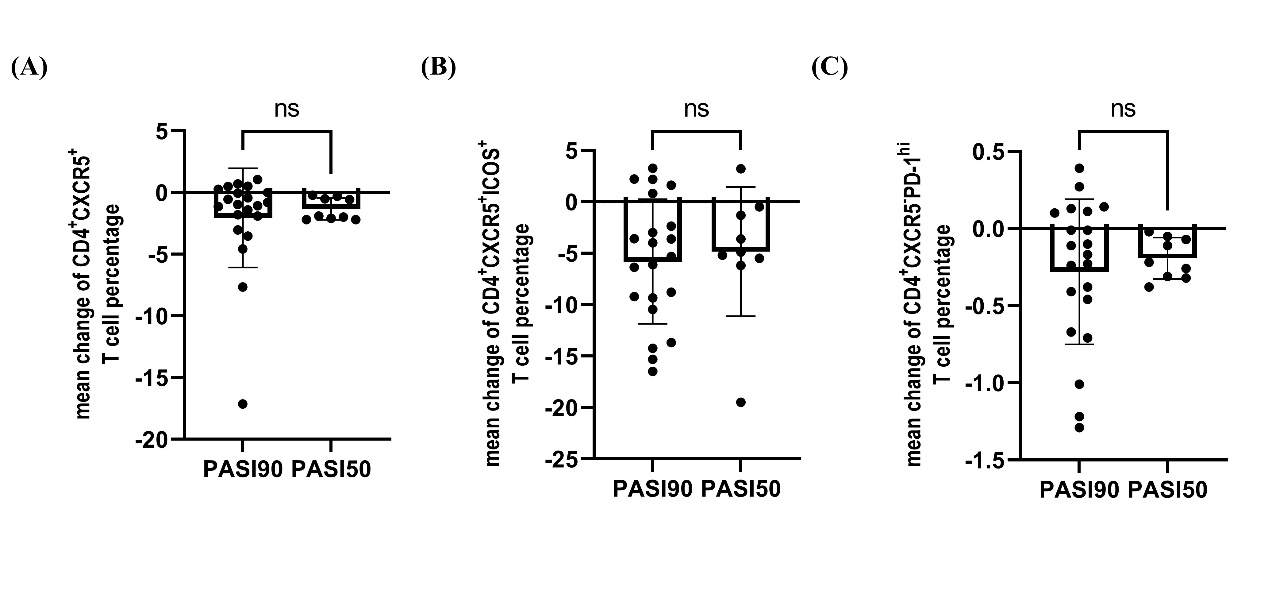
**

**Supplementary Figure 4.** **No significant differences in mean cell percentage changes were observed between complete responders (CRs) and partial responders (PRs).**  In total, 30 Ps patients achieved at least 50% improvement in Psoriasis Area Severity Index (PASI50) at 3 months after biologic therapy initiation. Twenty-one of 30 Ps patients achieved at least 90% improvement in PASI (PASI90) and 7 patients achieved cleared skin (100% improvement in PASI, PASI 100). Accordingly, patients were grouped in a PRs group (achieving at least PASI50, but not PASI90 or more, n=9) and a CRs group (achieving at least PASI90, including PASI100, n=21). Differences in mean values of changes at percentages (induced by anti-IL-17 biologic therapy) between different groups were not observed for any of the examined T cell subpopulations. **(A)** Box graphical representation showing no difference in mean value of percentage change between groups in CD4^+^CXCR5^+^ T cell subset. **(B)** Box graphical representation showing no difference in mean value of percentage change between groups in CD4^+^CXCR5^+^ICOS^+^ T cell subset. **(C)** Box graphical representation showing no difference in mean value of percentage change between groups in CD4^+^CXCR5^-^PD-1^hi^ T cell subset. ns p >0.05, by one-way ANOVA


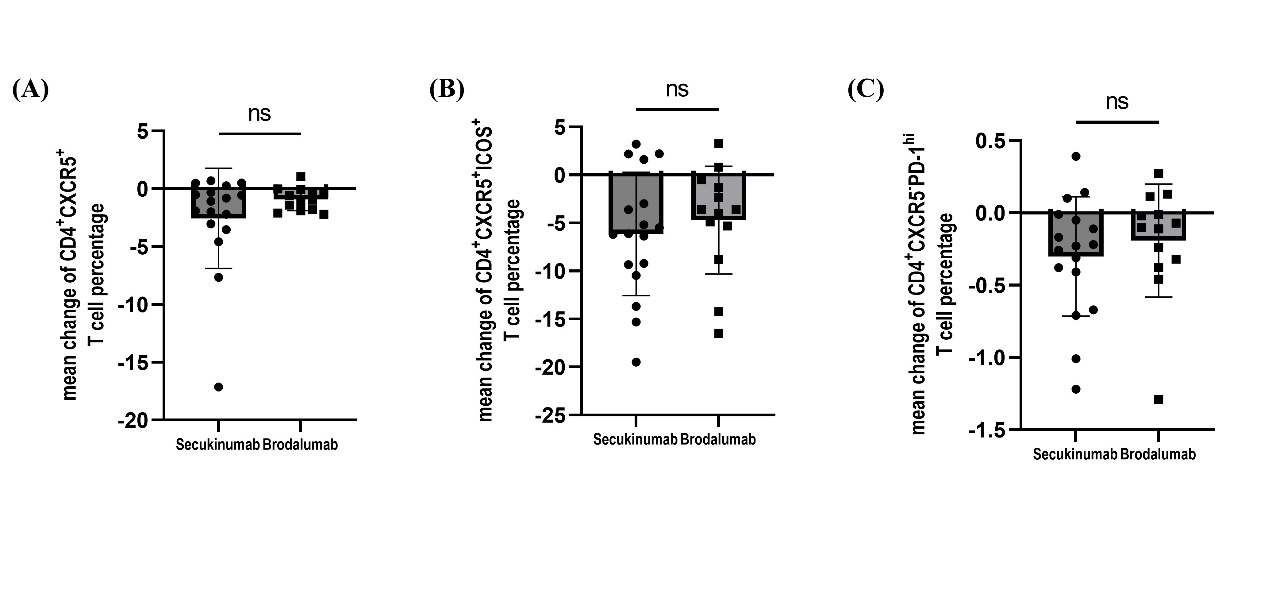
**Supplementary Figure 5.** **No significant differences in mean cell percentage changes were observed between secukinumab- or brodalumab-induced effects**. Of the 30 Ps patients, 17 were treated with secukinumab and 13 were treated with brodalumab up to the 3 month-timepoint assessment. No significant differences in mean cell percentage changes were observed between secukinumab- or brodalumab-induced effects. **(A)** Box graphical representation showing no difference in mean value of percentage change between secukinumab and brodalumab treated patients in CD4^+^CXCR5^+^ T cell subset. **(B)** Box graphical representation showing no difference in mean value of percentage change between secukinumab and brodalumab treated patients in CD4^+^CXCR5^+^ICOS^+^ T cell subset. **(C)** Box graphical representation showing no difference in mean value of percentage change between secukinumab and brodalumab treated patients in CD4^+^CXCR5^-^PD-1^hi^ T cell subset. ns p >0.05, * p ≤0.05, by unpaired t-test or Mann-Whitney test

**
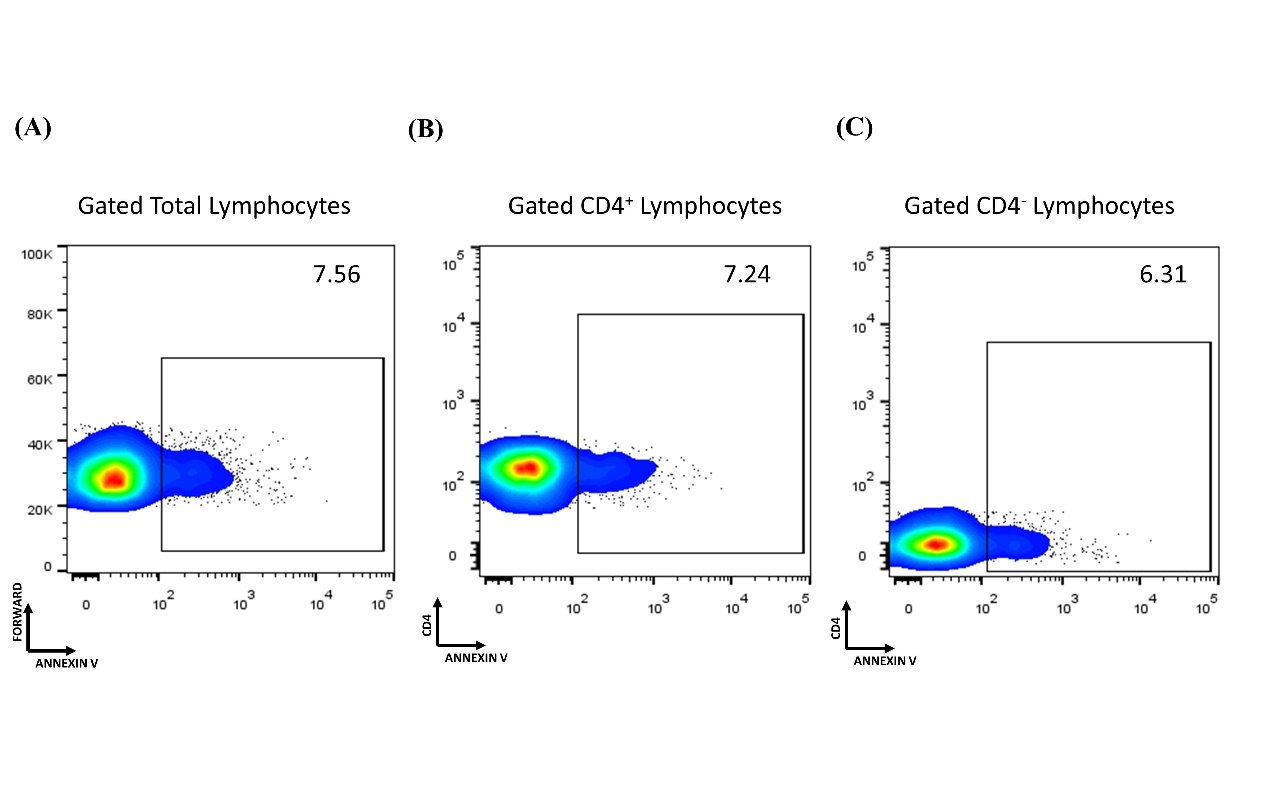
**

**Supplementary Figure 6.** **Thawing of PBMCs did not induce apoptosis of cells. (A)** Representative flow cytometry plot of cell viability assessment utilizing Annexin V staining. Total lymphocytes have been gated. **(B)** Representative flow cytometry plot of cell viability assessment utilizing Annexin V staining. CD4^+^ lymphocytes have been gated. **(C)** Representative flow cytometry plot of cell viability assessment utilizing Annexin V staining. CD4^-^ lymphocytes have been gated. Thawing did not affect viability nor induced apoptosis in significant numbers

**
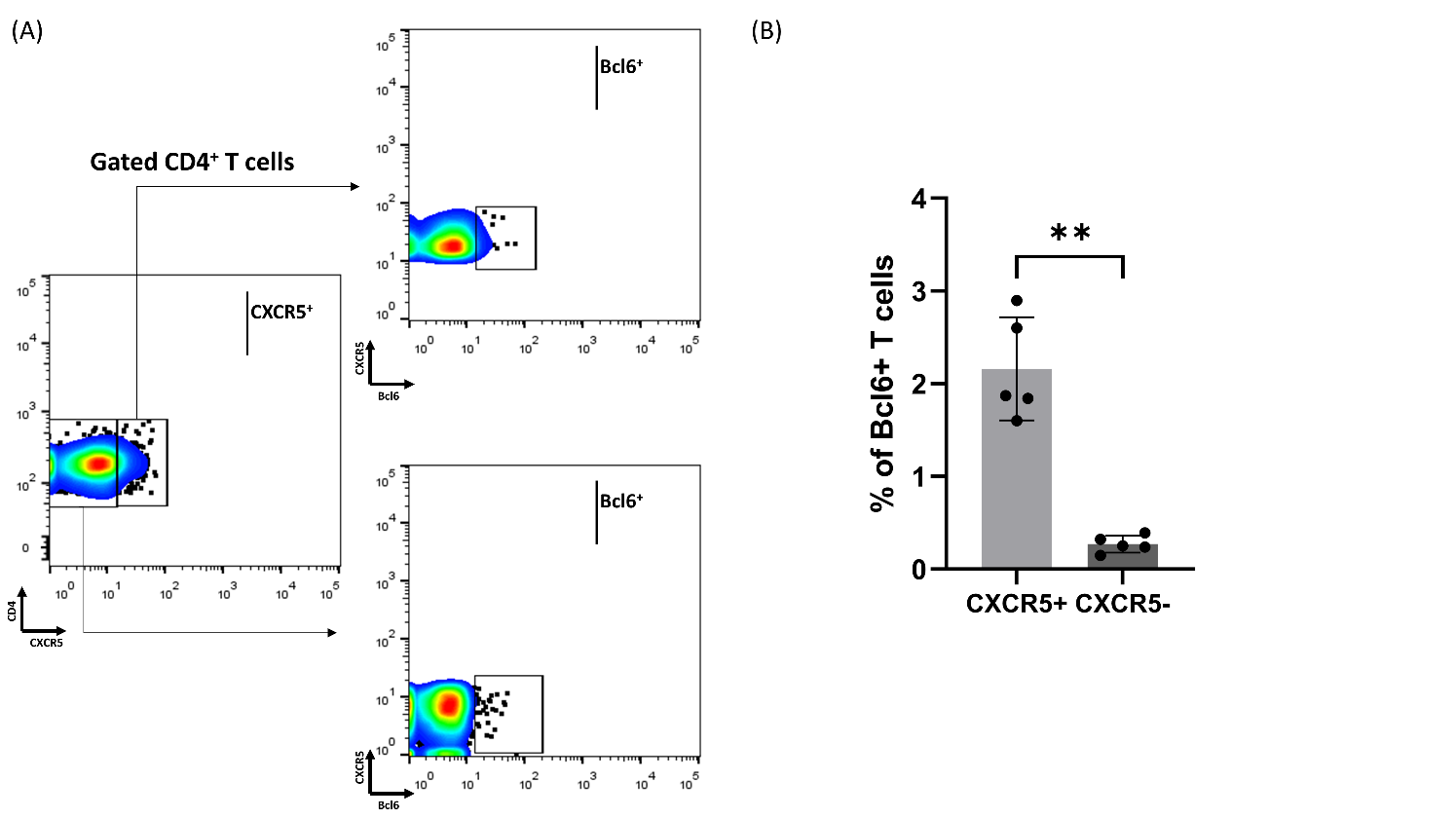
**

**Supplementary Figure 7.** **Bcl-6 expression significant differed between CD4+CXCR5+ and CD4+CXCR5- cells in periphery.** **(A)** Representative flow cytometry plot of cells expressing Bcl-6. CD4+ T cells have been gated. **(B)** Box graphical representation showing the significant difference. ** p ≤0.01, by paired t-test.

## Supplementary Tables

**Supplementary Table 1. List of reagents used in flow cytometry.**

| **Antibody** | **Fluorochrome** | **Source** | **Cat. No.** | **Clone** |
| --- | --- | --- | --- | --- |
| Mouse anti-human CXCR5 | FICT | BioLegend | 356914 | J252D4 |
| Mouse anti-human PD-1 | PE | BioLegend | 329906 | EH12.2H7 |
| Mouse anti-human CCR6 | PE | BioLegend | 353410 | G034E3 |
| Mouse anti-human CD3 | PerCP | BioLegend | 300326 | HIT3a |
| Mouse anti-human CD4 | Pe-Cy7 | BioLegend | 300512 | RPA-T4 |
| Armenian hamster anti-human/mouse/rat ICOS | APC-Cyanine7 | BioLegend | 313530 | C398.4A |

* FITC: fluorescein isothiocyanate; PE: phycoerythrin; PerCP: peridinin chlorophyll protein; Pe-Cy7: phycoerythrin-cyanine 7; APC-Cyanine7: allophycocyanin-cyanine7; Cat. No.: catalog number; BioLegend: BioLegend, San Diego, USA

**Supplementary Table 2. Setup of flow cytometer.**

| Instrument: | Guava EasyCyte 8 (Merck-Millipore, Burlington, USA) | | | | |
| --- | --- | --- | --- | --- | --- |
| Laser lines | 488 nm | | | | 642 nm |
| Emission filters | 525/30 | 583/26 | 695/50 | 785/70 | 785/70 |
| Fluorochromes | FITC | PE | PerCP | Pe-Cy7 | APC-Cyanine7 |

**Supplementary Table 3. Cell sub-population characterization by co-expression of surface markers.**

| **Phenotype** | **Cell population** |
| --- | --- |
| CD3^+^CD4^+^CXCR5^+^ | cTfh |
| CD3^+^CD4^+^ICOS^+^ | ICOS^+^ Th |
| CD3^+^CD4^+^PD-1^+^ | PD-1^+^ Th |
| CD3^+^CD4^+^CXCR5^+^ICOS^+^ | ICOS^+^-cTfh |
| CD3^+^CD4^+^CXCR5^+^PD-1^+^ | PD-1^+^-cTfh |
| CD3^+^CD4^+^CXCR5^+^ICOS^+^PD-1^+^ | ICOS^+^PD-1^+^-cTfh |
| CD3^+^CD4^+^CXCR5^-^PD-1^hi^ | cTph |

* cTfh: circulating follicular helper T; Th: T helper; cTph: circulating T peripheral helper

**Supplementary Table 4. Routine laboratory lymphocyte analysis of psoriasis patients (n=20) at baseline and at 3 months after biologic anti-IL17A therapy.**

|  | Timepoints | |
| --- | --- | --- |
|  | Baseline (10^9^/L) | Three months (10^9^/L) |
| Leukocyte Count | 7.83 ± 1.56 | 7.57 ± 1.69 |
| Lymphocytes | 2.15 ± 0.56 | 2.23 ± 0.72 |
| Monocytes | 0.57 ± 0.11 | 0.54 ± 0.11 |
| Eosinophiles | 0.21 ± 0.10 | 0.23 ± 0.11 |
| Basophiles | 0.03 ± 0.03 | 0.03 ± 0.02 |

* Values are listed as mean ± SD.
